# Defensive medicine among neurosurgeons in the Netherlands: a national survey

**DOI:** 10.1007/s00701-017-3323-9

**Published:** 2017-09-20

**Authors:** Sandra C. Yan, Alexander F. C. Hulsbergen, Ivo S. Muskens, Marjel van Dam, William B. Gormley, Marike L. D. Broekman, Timothy R. Smith

**Affiliations:** 1 Computational Neuroscience Outcomes Center (CNOC), Department of Neurosurgery, Brigham and Women’s Hospital, Harvard Medical School, Boston, MA USA; 2 Department of Neurosurgery, Rudolf Magnus Institute for Neuroscience, University Medical Center Utrecht, Heidelberglaan 100, 3584 CX Utrecht, The Netherlands; 3 Department of Intensive Care Medicine, University Medical Center Utrecht, Utrecht, The Netherlands; 4 Department of Neurology, Massachusetts General Hospital, Harvard Medical School, Boston, MA USA

**Keywords:** The Netherlands, Medico-legal environment, Neurosurgery, Defensive medicine, Liability, Malpractice

## Abstract

**Objective:**

In defensive medicine, practice is motivated by legal rather than medical reasons. Previous studies have analyzed the correlation between perceived medico-legal risk and defensive behavior among neurosurgeons in the United States, Canada, and South Africa, but not yet in Europe. The aim of this study is to explore perceived liability burdens and self-reported defensive behaviors among neurosurgeons in the Netherlands and compare their practices with their non-European counterparts.

**Methods:**

A survey was sent to 136 neurosurgeons. The survey included questions from several domains: surgeon characteristics, patient demographics, type of practice, surgeon liability profile, policy coverage, defensive practices, and perception of the liability environment. Survey responses were analyzed and summarized.

**Results:**

Forty-five neurosurgeons filled out the questionnaire (response rate of 33.1%). Almost half (*n* = 20) reported paying less than 5% of their income to annual malpractice premiums. Nearly all respondents view their insurance premiums as a minor or no burden (*n* = 42) and are confident that in their coverage is sufficient (*n* = 41). Most neurosurgeons (*n* = 38) do not see patients as “potential lawsuits”.

**Conclusions:**

Relative to their American peers, Dutch neurosurgeons view their insurance premiums as less burdensome, their patients as a smaller legal threat, and their practice as less risky in general. They are sued less often and engage in fewer defensive behaviors than their non-European counterparts. The medico-legal climate in the Netherlands may contribute to this difference.

## Introduction

Defensive medicine (DM) is a departure from standard medical practices out of a fear of litigation [19]. There are two types of defensive medicine: positive DM is the practice of prescribing unnecessary, additional medical treatment out of a fear of lawsuits, and negative DM is avoiding high-risk procedures, which could compromise clinical decision-making [16]. DM is especially prevalent in high-risk fields, such as neurosurgery, and has been estimated to contribute as much as $60.2 billion annually to healthcare expenditures in the United States alone [8, 12, 17].

Besides an extra financial burden, DM can also result in direct or indirect dangers for individual patients. Positive DM can lead to exposure to unnecessary procedures that come with additional risks. An example of tests that are commonly performed in the context of DM are CT scans. However, it has been shown that these can cause long-term morbidity due to radiation exposure [4, 5]. On the other hand, negative DM can lead to a local unavailability of critical care. A drastic example of this is Chester County, PA, USA, where at least two patients died during an hour-long ambulance trip to the next county. A nearer hospital was not able to help them because of a lack of neurotrauma surgeons due to the local legal climate [12].

A previous survey performed in the United States demonstrated a correlation between medico-legal risk environment and self-reported defensive behaviors in neurosurgeons [14]. Gaining oversight of the extent of DM can help devise strategies to tackle its negative consequences. Comparing defensive climates across countries with different health care and liability systems may reveal strengths and weaknesses on both sides and provide perspectives towards a more balanced liability climate.

The Netherlands is frequently ranked as having one of the best healthcare systems in Europe, with mandatory universal health insurance and competition among private health insurers [20]. Furthermore, the Dutch medico-legal climate differs considerably from its US equivalent; most notably, it actively offers patients several alternatives to lawsuits in case of complaints. One of these is the disciplinary board or medical legal board: a special Dutch law court that deals exclusively with cases of allegations of malpractice. It can impose punitive measures on health professionals, but it does not grant financial compensation to patients [2].

In this study, we examine the perceived liability burden among Dutch neurosurgeons and their defensive behaviors in the context of the Dutch medico-legal system and their perception of the American medico-legal climate.

## Methods

An online survey containing 55 questions on neurosurgeons’ perceptions of the medico-legal landscape and their practice of DM was sent to 136 Dutch neurosurgeons in May 2016. These surgeons were reached through the Dutch Neurosurgical Society (Nederlandse Vereniging voor Neurochirurgie), of which all Dutch neurosurgeons are a member. The questionnaire was developed with input from various neurosurgical associations, including the American Association of Neurological Surgery, the American Board of Neurological Surgery, and the Congress of Neurological Surgery, and has been used in previous defensive medicine surveys in the USA [14, 15]. Dutch colleagues translated the questions into Dutch and culturally customized them to neurosurgical and legal practices specific to the Netherlands. However, scales for financial questions were not altered to maintain maximum comparability between surveys from different countries. The questionnaire was re-sent twice after initial distribution to maximize response rate.

The survey included questions in eight domains: surgeon characteristics, patient demographics, surgeon practice type, insurance information, surgeon liability profile, policy coverage, defensive behaviors, and perception of the liability environment. There was a possibility to select multiple answers for all questions. The survey was anonymous and respondents had the option of skipping questions. Data analysis was performed using IBM® SPSS® version 23 (IBM SPSS Inc., Armonk, NY, USA). Basic demographics were summarized using counts and percentages for nominal variables and means, medians, and standard deviations/ranges for continuous variables.

## Results

Forty-five of the 136 Dutch neurosurgeons completed the questionnaire, for a response rate of 33.1%. Thirty-nine respondents were male and four were female, while two preferred not to tell. Eleven neurosurgeons were younger than 40 years old, and one respondent was doing a fellowship. In the Netherlands, fellows are neurosurgeons who have finished their residency but receive additional training in a specific subfield of neurosurgery while not being in a permanent position. Respondents most often performed between 200 and 300 surgeries each year (*n* = 17), with none operating on fewer than 100 cases annually and four operating on more than 500 cases annually. Eight neurosurgeons have operated on 1001–2000 cases in their lifetime, while 11 have operated on between 4001 and 5000 cases, and nine have operated on between 5001 and 10,000 cases (Table 1).

**Table 1:** Basic characteristics of respondents

Characteristic	*N* (%)*
Sex	
Male	39 (86.7)
Female	4 (8.8)
Age, years	
30–39	11 (24.4)
40–49	15 (33.3)
50–59	15 (33.3)
60–69	2 (4.4)
70–79	0 (0.0)
> 79	1 (2.2)
Clinical status	
In fellowship	1 (2.2)
< 5 years in practice	7 (15.6)
5–10 years in practice	14 (31.1)
10–20 years in practice	12 (26.7)
20–30 years in practice	8 (17.8)
> 30 years in practice	3 (6.7)
Number of annual operative cases	
< 50 cases	0 (0.0)
50–100	0 (0.0)
100–200	8 (17.8)
200–300	17 (37.8)
300–400	8 (17.8)
400–500	7 (15.6)
> 500	4 (8.8)
Number of lifetime operative cases	
< 500 cases	1 (2.2)
501–1000	0 (0.0)
1001–2000	8 (17.8)
2001–3000	4 (8.8)
3001–4000	6 (13.3)
4001–5000	11 (24.4)
5001–10,000	9 (20.0)
> 10,000	6 (13.3)

*Sums of percentages lower or higher than 100% are the result of respondents skipping questions or selecting multiple answers, respectively

The majority of neurosurgeons (*n* = 31) practice in an academic setting, while 14 practice in a general community setting. Another 18 are in a group practice. Since many Dutch neurosurgeons work in an academic/community setting and in group/solo practice simultaneously, for example part-time in an academic hospital and part-time in a community hospital, these percentages amount to over 45 (i.e., 100% of respondents). The majority of respondents (*n* = 23) are generalists, while 17 practice cerebrovascular surgeries, 27 operate on brain tumors, and another 23 practice spine surgery. Once again, since some surgeons practice several subspecialties, the sum of these numbers is higher than 45. Most respondents (*n* = 23) reported having more than ten neurosurgical colleagues in their practices (Table 2).

**Table 2:** Practice characteristics

Characteristic	*N* (%)*
Type of practice	
Pediatrics	9 (20.0)
Functional	3 (6.7)
Cerebrovascular	17 (37.8)
Tumor	27 (60.0)
Spine	23 (51.1)
Trauma	6 (13.3)
Generalist	23 (51.1)
Setting	
Academic	31 (68.9)
General community	14 (31.1)
Group practice	18 (40.0)
Solo: private practice	3 (6.7)
Number of neurosurgical colleagues	
Zero	0 (0.0)
1–2	0 (0.0)
3–5	8 (17.8)
6–10	15 (33.3)
> 10	23 (51.1)

*Sums of percentages lower or higher than 100% are the result of respondents skipping questions or selecting multiple answers, respectively

Concerning insurance-related questions, a third of respondents (*n* = 15) reported paying less than €1000 ($1107) annually for liability insurance, while five reported paying between €1001 ($1108) and €5000 ($5535); interestingly, no respondent reported paying more than €20,000 ($22,140) for annual malpractice liability. Almost half (*n* = 20) of respondents reported that less than 5% of their annual incomes goes towards paying malpractice premiums, and nearly all (*n* = 42) reported that their insurance premiums were a minor burden or no burden at all. Fourteen reported that there has not been a significant change in their liability premiums in the past 3 years, while four reported there has been an increase, and one reported there has been a decrease in liability premiums. Approximately two-thirds (*n* = 30) believe that an appropriate annual malpractice premium for neurosurgeons should be less than €5000 ($5535), and only one believes an appropriate premium should be greater than €50,000 ($55,350). Of note, 41 respondents are confident that their insurance policies provide adequate coverage (Tables 3 and 4).

**Table 3:** Insurance coverage

Characteristic	*N* (%)*
Mandated minimum level of coverage	
< €250,000	2 (4.4)
€250,000–€500,000	1 (2.2)
€500,000–€1 million	0 (0.0)
€1–€2 million	1 (2.2)
€2–€3 million	1 (2.2)
€3–€4 million	0 (0.0)
€4–€5 million	0 (0.0)
> €5 million	0 (0.0)
Not required	4 (8.8)
My institution provides coverage	28 (62.2)
I do not know	8 (17.8)
Annual cost of liability insurance	
< €1000	15 (33.3)
€1001–€5000	5 (11.1)
€5001–€10,000	1 (2.2)
€10,001–€15,000	0 (0.0)
€15,001–€20,000	1 (2.2)
€20,001–€25,000	0 (0.0)
€25,001–€30,000	0 (0.0)
> €30,000	0 (0.0)
I do not know	23 (51.1)
Percentage of annual income going to malpractice premiums	
< 5%	20 (44.4)
6–10%	2 (4.4)
10–19%	1 (2.2)
20–29%	0 (0.0)
30–39%	0 (0.0)
40–49%	0 (0.0)
50–59%	0 (0.0)
> 60%	0 (0.0)
I do not know	22 (48.9)
Overall burden of liability insurance premiums	
Not a burden	31 (68.9)
A minor burden	11 (24.4)
A major burden	2 (4.4)
An extreme burden	0 (0.0)

*Sums of percentages lower or higher than 100% are the result of respondents skipping questions or selecting multiple answers, respectively

**Table 4:** Insurance coverage (continued)*

Characteristic	*N* (%)
Policy payout per occurrence	
< €1 million	4 (8.8)
€1000,001–€3 million	3 (6.7)
> €3 million	1 (2.2)
I do not know	36 (80.0)
Policy payout maximum	
< €1 million	2 (4.4)
€1000,001–€3 million	4 (8.8)
€3000,001–€5 million	3 (6.7)
> €5 million	2 (4.4)
I do not know	34 (75.6)
Change in liability premiums in the past 3 years	
> 25% increase	1 (2.2)
> 10% increase	3 (6.7)
No significant change	14 (31.1)
> 10% decrease	0 (0.0)
> 25% decrease	1 (2.2)
I do not know	26 (57.8)
Change in average schedule of benefits in the past 3 years	
> 25% increase	0 (0.0)
> 10% increase	2 (4.4)
No significant change	8 (17.8)
> 10% decrease	1 (2.2)
> 25% decrease	3 (6.7)
I do not know	31 (68.9)
Appropriate annual malpractice premium for neurosurgeons	
< €5000	30 (66.7)
€6000–€10,000	3 (6.7)
€11,000–€20,000	3 (6.7)
€21,000–€30,000	2 (4.4)
€31,000–€40,000	0 (0.0)
€41,000–€50,000	1 (2.2)
> €50,000	1 (2.2)
Confidence that insurance policy provides adequate coverage	
Very confident	22 (48.9)
Somewhat confident	19 (42.2)
Not very confident	1 (2.2)
Not at all confident	0 (0.0)

*Sums of percentages lower or higher than 100% are the result of respondents skipping questions or selecting multiple answers, respectively

Regarding complaints by patients, 12 respondents did not have any filed against them, legal or otherwise, in the previous 3 years. Twenty-three have faced 1–2 and nine have faced 3–4 complaints, while none have had to deal with more. A large majority of neurosurgeons indicated their institutions provide a complaint mediator (*n* = 34) and/or a complaint committee (*n* = 41) to deal with medical complaints. Another three indicated their institutions provide other services, including staff lawyers.

Few respondents had experiences with complaints filed via legal procedures. Thirty-two have never appeared before the medical disciplinary board. Moreover, 41 have never been sued in civil court. Overall, 32 respondents have never had any financial claims made against them in their careers, whether in court or via extrajudicial complaints committees (Table 5).

**Table 5:** Previous complaints and lawsuits

Characteristic	*N* (%)*
Complaints in past 3 years	
None	12 (26.7)
1–2	23 (51.1)
3–4	9 (20.0)
5–6	0 (0.0)
7–8	0 (0.0)
9–12	0 (0.0)
13–15	0 (0.0)
> 15	0 (0.0)
Services provided by institution to handle medical complaints	
None	0 (0.0)
A complaint mediator	34 (75.6)
A complaint committee	41 (91.1)
I do not know	0 (0.0)
Other	3 (6.7)
How filed complaints have been handled	
Via a mediator provided by my institution	13 (28.9)
Via my institution’s complaint committee	16 (35.6)
Via an external complaint institution	1 (2.2)
Via the medical legal board	4 (8.8)
In court	0 (0.0)
Not applicable	9 (20.0)
Other	1 (2.2)
Appearances before the medical legal board	
Never	32 (17.1)
Once	9 (20.0)
Twice	3 (6.7)
3–5 times	1 (2.2)
> 5 times	0 (0.0)
Number of adverse judgments made if appeared before the medical legal board	
Zero	8 (17.8)
One	3 (6.7)
Two	0 (0.0)
3–5	1 (2.2)
>5	0 (0.0)
N/A	31 (68.9)
Type of adverse judgment	
Warning	3 (6.7)
Reproach	2 (4.4)
Fine	0 (0.0)
Suspension	0 (0.0)
Termination of medical license	0 (0.0)
N/A	40 (88.9)
Urging patients to go to the medical legal board to prevent being sued in civil court	
No	44 (97.8)
Yes	1 (2.2)
Financial claims in past 3 years	
Never	37 (82.2)
1–2	7 (15.6)
3–4	0 (0.0)
5–6	0 (0.0)
7–8	0 (0.0)
9–12	1 (2.2)
13–15	0 (0.0)
> 15	0 (0.0)
Financial claims in career	
Never	32 (71,1)
1–2	8 (17.8)
3–4	4 (8.8)
5–6	0 (0.0)
7–8	0 (0.0)
9–12	1 (2.2)
13–15	0 (0.0)
> 15	0 (0.0)
Financial claims in civil court made after medical legal board judgment	
Never	18 (40.0)
Once	0 (0.0)
Twice	0 (0.0)
3–5 times	0 (0.0)
> 5 times	0 (0.0)
I do not know	1 (2.2)
N/A	26 (57.8)
Nature of medical legal board judgment if financial claims made after medical legal board judgment	
Honoring the complaint	2 (4.4)
Dismissal of the complaint	1 (2.2)
N/A	41 (91.1)
Number of lifetime adverse settlements/judgments made if sued in civil court	
None	8 (17.8)
1–2	2 (4.4)
2–3	0 (0.0)
4–5	1 (2.2)
6–7	0 (0.0)
> 7	0 (0.0)
N/A	34 (75.6)
How recently sued in civil court	
< 3 years ago	2 (4.4)
> 3 years ago	1 (2.2)
Both	1 (2.2)
Never been sued	41 (91.1)

*Sums of percentages lower or higher than 100% are the result of respondents skipping questions or selecting multiple answers, respectively

Only five respondents believed there was a medical liability crisis in their area, and only three would have chosen a different specialty if the current medical liability situation was present when they were starting their careers. An overwhelming majority (*n* = 38) do not view their patients as “potential lawsuits”. Among defensive behaviors, ordering additional imaging was found to be the most prevalent, with 29 respondents reporting doing so. Thirteen reported referring patients, five ordered laboratory tests, and four reported prescribing medications. Of note, several respondents reported not engaging in any defensive behaviors (Tables 6 and 7).

**Table 6:** Perception of liability crises

How would you respond to each of the following statements?*				
	There is a medical liability crisis in my area.	Medical liability affects where I practice neurosurgery geographically	If the current medical liability situation was present when I was choosing neurosurgery as a career, I would have chosen a different specialty.	I see each patient as a potential lawsuit
Strongly agree	0 (0.0)	2 (4.4)	1 (2.2)	1 (2.2)
Agree	5 (11.1)	9 (20.0)	2 (4.4)	2 (4.4)
Neutral	13 (28.9)	13 (28.9)	6 (13.3)	2 (4.4)
Disagree	18 (40.0)	10 (22.2)	15 (33.3)	14 (31.1)
Strongly disagree	8 (17.8)	9 (20.0)	20 (44.4)	24 (53.3)

*Sums of percentages lower or higher than 100% are the result of respondents skipping questions or selecting multiple answers, respectively

**Table 7:** Defensive behaviors in respondents

Characteristic			*N* (%)*	
Actions performed solely to minimize the risk of a lawsuit				
Ordered laboratory tests			5 (11.1)	
Referred patients			13 (28.9)	
Prescribed medications			4 (8.8)	
Suggested a procedure			3 (6.7)	
Ordered imaging			29 (64.4)	
Other			3 (6.7)	
Frequency of actions performed by respondents for defensive purposes*				
	Order extra lab tests	Order extra imaging	Obtain previous consultations	Refer to colleagues
Always	0 (0.0)	0 (0.0)	0 (0.0)	0 (0.0)
Very often	1 (2.2)	5 (11.1)	1 (2.2)	1 (2.2)
Sometimes	6 (13.3)	17 (37.8)	13 (28.9)	6 (13.3)
Rarely	19 (42.2)	18 (40.0)	22 (48.9)	22 (48.9)
Never	19 (42.2)	5 (11.1)	8 (17.8)	16 (35.6)

*Sums of percentages lower or higher than 100% are the result of respondents skipping questions or selecting multiple answers, respectively

When asked which procedures they provided they would consider high-risk, 18 neurosurgeons indicated cranial trauma cases and 18 indicated subdural evacuations. Seventeen responded they would consider tumor resections to be high-risk, with another nine listing other procedures, including degenerative spinal surgery. Only one respondent reported they were considering retirement due to the medico-legal climate. Five surgeons answered affirmatively when asked if they had ever received neurosurgical training in the United States. Four of these reported that neurosurgical practice is more defensive in the USA than in the Netherlands, partially due to the surrounding institutional culture. A majority of respondents (*n* = 32) believe that defensive medicine is a problem in the United States, whereas only 12 believe that defensive medicine is a problem in the Netherlands. Thirty-three think that medical liability increases the cost of practicing neurosurgery in the USA as compared to the Netherlands. Of note, several respondents made comments that while defensive medicine is currently not a pressing issue among Dutch neurosurgeons, they fear that it will become more problematic in the future if the Netherlands embraces a medical-legal environment in which civil claims are more easily filed, as is the case in the USA according to respondent’s perception (Tables 8 and 9).

**Table 8:** Influence of malpractice climate on surgeon behavior

Characteristic	*N* (%)*
High-risk services/procedures	
Shunts (pediatrics)	13 (28.9)
Spine trauma	16 (35.6)
Workers’ compensation injury	9 (20.0)
Head trauma	18 (40.0)
Subdural evacuation	18 (40.0)
Epidural evacuation	15 (33.3)
Aneurysm coiling	1 (2.2)
Aneurysm clipping	12 (26.7)
Tumor resection	17 (37.8)
Other	9 (20.0)
Reasons for discontinuing high-risk services/procedures	
Liability	0 (0.0)
Technical skill involved	0 (0.0)
Dislike	0 (0.0)
Changing practice	3 (6.7)
N/A	42 (93.3)
Other	1 (2.2)
Number of trauma patients seen per week	
> 15	1 (2.2)
10–15	0 (0.0)
5–10	2 (4.4)
3–5	9 (20.0)
< 3	29 (64.4)
Do not see trauma patients	2 (4.4)
Thinking about retirement due to malpractice climate	
Yes	1 (2.2)
No	5 (11.1)
N/A	37 (82.2)
Moved to another province due to medical liability concerns	
Yes	1 (2.2)
No	42 (93.3)
Adopting asset protection strategies in the next two years due to malpractice liability concerns	
Yes	6 (13.3)
No	32 (71.1)
N/A	5 (11.1)

*Sums of percentages lower or higher than 100% are the result of respondents skipping questions or selecting multiple answers, respectively

**Table 9:** Perception of defensive climate in the United States

Characteristic	*N* (%)*
Practiced/Trained in the USA	
Yes	5 (11.1)
No	38 (84.4)
Practiced more defensively in the USA if trained there	
Yes	4 (8.8)
No	1 (2.2)
I do not know	1 (2.2)
N/A	36 (80.0)
Reasons for practicing more defensively in the USA	
Fear of a lawsuit	2 (4.4)
Influence of mentors	2 (4.4)
Adopted the surrounding institutional “culture”	3 (6.7)
I do not know	0 (0.0)
N/A	35 (77.8)
Other	0 (0.0)
Think defensive medicine is a problem in the USA	
Yes	32 (71.1)
No	0 (0.0)
I do not know	10 (22.2)
Think defensive medicine is a problem in the Netherlands	
Yes	12 (26.7)
No	25 (55.6)
I do not know	6 (13.3)
Think medical legal liability increases the cost of practicing neurosurgery in the USA compared to the Netherlands	
Yes	33 (73.3)
No	2 (4.4)
I do not know	8 (17.8)

*Sums of percentages lower or higher than 100% are the result of respondents skipping questions or selecting multiple answers, respectively

## Discussion

Our study is the first to report the results of a survey on defensive medicine among neurosurgeons from a European country. The results show a positive situation: Dutch neurosurgeons indicate that they experience light burdens from insurance premiums, are infrequently confronted with financial claims, and most defensive behaviors, such as ordering extra lab tests, are rarely practiced.

Several factors may contribute to the fact that DM is relatively uncommon in the Netherlands. The low reported insurance premiums and high confidence of respondents in their insurer’s coverage indicate that little financial incentives exist for surgeons to deviate from standard, non-defensive practice. Moreover, in the subgroup of surgeons that had practiced in the United States, institutional culture was the most cited reason for practicing more defensive medicine in US clinics. It is possible that, while initially motivated by financial pressure, DM has partly become ingrained in the institutional culture of some clinics. DM has thus become a cultural as well as a financial phenomenon. A third explanation for the favorable DM climate in the Netherlands is the Dutch medico-legal system. This protects physicians against financial claims by offering dissatisfied patients several alternatives to filing civil lawsuits.

### The Dutch medico-legal system

The medico-legal climate and healthcare system in the Netherlands differ from those in the United States. The Netherlands has mandatory basic health insurance and government-sponsored long-term care. The Dutch healthcare system manages patient complaints regarding their physicians in a multi-step approach [2, 3, 10]. Patients wishing to share dissatisfaction with their doctors can turn to a complaints mediator provided by the hospital. A complaints mediator is an easily approachable hospital employee whose primary responsibility is to establish an agreement and reconciliation between the patient and the physician. A disagreement can arise from various factors, including when patients feel their input into, and concerns regarding, their treatment options are not being taken seriously. If the complaints mediator fails to remedy patients’ dissatisfaction to their content, the next step would be going to a hospital’s complaints committee. This is an official institution within the hospital’s management structure that handles complaints submitted by letter in a formal way. If the committee rules the complaint is justified, it will start an investigation and possibly impose measures upon a department to prevent future repetition of the mistake. The committee will also submit a final summary of its complaint findings to the hospital’s board of directors. Since January 1, 2017, a third step for a patient would be to turn to a conciliation board (Geschillencommissie), an independent institution that can investigate complaints and also impose measures on caregivers. These boards have replaced local complaint committees on January 1, 2017, under the new Healthcare Quality, Complaints, and Disputes Act (Wet Kwaliteit, Klachten en Geschillen in de Zorg) [9, 10].

Aside from these options, patients may choose to turn to the disciplinary board. Disciplinary law is a special branch of the Dutch judicial system that deals specifically with cases of malpractice by physicians and other healthcare professionals, such as pharmacists and dentists. The disciplinary board consists of both medical and legal professionals and is independent from any health-related institution; rather, it is a part of the judicial power. The board focuses on the healthcare provider and can impose measures including a warning, a reprimand, a fine of up to €4500 ($4981.50), a temporary suspension, or a permanent revocation of a medical license depending on the severity of the misconduct [2].

For patients seeking financial compensation, there are several options. The most straightforward would be for patients to approach the hospital’s insurance company, present their cases, and request compensation. If the insurance company concludes that the financial claim is legitimate and proportional, it will compensate. In practice, about a third of applications are approved [2]. A verdict from the disciplinary board may help the patient when applying; the committee may even advise patients to go to the insurance company. If the insurance company fails to grant any financial compensation, the second step would be turning to the conciliation board as described above; the board has the authority to grant compensation of up to €25,000 ($27,675) [10]. The other option would be to sue in civil court.

The aforementioned steps do not have to be followed in the described order; a patient is free to turn to any of the institutions at any time. Patients may have various motivating factors for going to any of these institutions, including a desire for official recognition of a complaint, financial compensation, prevention of future instances of similar malpractice, or even retribution. It should be noted that any of these motivations can be fulfilled by options that require significantly less time, money, and effort than suing in civil court.

Several routes might be taken simultaneously; a patient could go to the disciplinary board to prevent future malpractice and to civil court for financial compensation. This has happened at least once to a neurosurgeon [6], who was acquitted by the disciplinary board but condemned in civil court. Ultimately, the Supreme Court (Hoge Raad) ruled that civil court rulings have to meet an increased burden of evidence if they wish to deviate from disciplinary board decisions, creating legal precedent protecting physicians [3].

This multi-step approach may play a role in the fact that our respondents reported an average of only 0.34 lawsuits in their entire careers, with 41 neurosurgeons reporting to have never been sued. Another reason for the infrequent lawsuits could be the fact that in Dutch civil court, the costs of the lawsuit process, as well as the legal fees of the winning party, have to be paid by the losing party if the judge decides so [11]. This may serve as a deterrent for filing frivolous lawsuits.

### Implications of survey results

In our analysis, we have demonstrated that Dutch neurosurgeons who were sued in the past may be more likely to practice defensive medicine in several ways, most notably by ordering extra imaging. This increase in defensive behavior is seen mainly after civil court claims and to a much lesser degree after appearance before the disciplinary board. This could suggest that alternatives to civil financial claims might be able to limit the practice of defensive medicine.

A previous report by Studdert et al. demonstrated that physicians’ level of confidence in their insurance coverage and their perception of the burden of their malpractice premiums were important predictors of defensive behaviors [17]. Our survey showed that only two neurosurgeons view their insurance premiums as burdensome (Table 3) and that 22 were “very confident” that their insurance policies provided adequate coverage (Table 4). Accordingly, one-third of Dutch neurosurgeons pay less than €1000 ($1107) in annual malpractice insurance premiums, and almost half reported that their malpractice premiums were less than 5% of their annual incomes (Table 3). In terms of actions that may influence defensive behavior, only three Dutch neurosurgeons in our survey viewed their patients as “potential lawsuits” (Table 6). This is in stark contrast to the United States, where about 70% of neurosurgeons indicated they lack confidence in their insurance coverage, even though they pay as much as 15% of their annual incomes on insurance premiums. Accordingly, 75% of American neurosurgeons viewed their patients as “potential lawsuits”. The results from the American and Dutch surveys combined support Studdert’s conclusions, as they indicate both a lower perceived burden and more restricted practice of DM in the Netherlands when compared to the United States [14, 17].

In the United States, defensive medicine practices predominate in high-risk specialties, such as neurosurgery and orthopedic surgery. A previous study looked at the largest physician-owned mutual insurance company in the United States and found that over a 5-year period, more than $600,000 was paid per neurosurgeon, making neurosurgery the most vulnerable specialty. This amount was double that of the next most vulnerable specialty, obstetrics. Additionally, roughly half of the neurosurgeons reported being sued at least once over that 5-year period, with roughly 25% having two suits filed against them [13]. Furthermore, in the USA, unwarranted lawsuits are frequently filed; a previous study found that 85% of lawsuits against neurosurgeons in a tertiary academic center were because the intended benefits of surgery were not seen despite the uneventful operations and were eventually withdrawn [7, 16, 18]. These studies demonstrate why neurosurgeons in the United States pay high malpractice premiums, with neurosurgeons from some US states paying as much as $400,000 annually in insurance premiums [15]. This is in stark contrast to the Netherlands, where most neurosurgeons pay less than €1000 ($1107).

Perhaps most interesting is Dutch neurosurgeons’ perception of defensive medicine in the United States. Four out of five respondents who practiced in the USA reported practicing more defensively than they would in the Netherlands, most often citing adoption of institutional culture as an explanation for this difference. Furthermore, the majority of Dutch neurosurgeons believe liability increases the cost of neurosurgical practice in the USA, and that DM is a significant problem in the USA (Table 9). However, since neurosurgeons are not legal experts, and individual perceptions are subjective, these particular responses should not be seen as an objective assessment of the US defensive medicine climate. Rather, these reflect the perception of the US climate among Dutch neurosurgeons.

Likewise, several remarks submitted by respondents suggest the perception and concern among some that the Dutch DM situation is gradually shifting towards the US’s. In particular, parts of the recently passed Healthcare Quality, Complaints, and Disputes Act have not been received entirely positively by the Dutch medical community. It is believed the introduction of low-effort financial claims through the conciliation board will increase the frequency of patients filing such claims and subsequently increase physicians’ DM practices. In debates revolving around this topic, the “American situation” is sometimes referred to as a negative outcome that should be avoided [1]. It remains to be seen what effect this law will have on physician behaviors several years down the road.

For Dutch neurosurgeons, oversight of the medico-legal system and the defensive climate both in the Netherlands and in other countries are essential for debate between all involved parties. Clinical decision-making, including defensive medicine, may be influenced by various incentives, such as legal, financial, and cultural motives. Insight into these motives is ultimately key to optimize clinical decision-making to ensure the best patient care.

### Limitations and future research

There are several limitations to our study. First of all, we were not able to raise the response rate of our survey above 33%, despite re-distributing the questionnaire several times. This could have resulted in a cross section of surgeons that is not fully representative of the entire Dutch neurosurgical community. For instance, surgeons with a large amount of complaints made against them or frequent appearances before the disciplinary board may perceive this as shameful and be disinclined to complete even an anonymous survey on this subject, which may have resulted in a selection bias. Respondents may also be less eager to openly admit that actions were taken against them, which may result in a reporting bias. Some questions had particularly low response rates because they were only applicable to particular subsets of respondents (e.g., only five surgeons had practiced in the USA), which may limit their implications. This questionnaire did not inquire whether surgeons performed exclusively cranial or spinal operations, which may be a crucial factor determining DM due to the difference in nature of the procedures. The differences among patients, procedures, and outcomes related between cranial and spinal neurosurgery may result in different forms of DM.

Physicians’ perceptions and beliefs play an important role in defensive medicine, but are subjective. These are, therefore, difficult to measure and should not be viewed as objective indicators of reality. Lastly, many factors may contribute to defensive behaviors among neurosurgeons, e.g., personal experience, confidence, and risk perception.

In future studies, it may be interesting to send out a similar questionnaire to European Association of Neurosurgical Societies (EANS) members to evaluate the DM climate on a European scale. However, differences in local legislations may contribute greatly to DM and make results hard to compare.

## Conclusions

Dutch neurosurgeons generally report their liability situation to be favorable. They rarely view their insurance premiums as burdensome or their patients as “potential lawsuits”. Both experience with legal procedures and defensive behaviors are relatively rare among respondents, especially when compared to their American colleagues.

## Abbreviations

DM: Defensive medicine
OR: Odds ratio
